# THE MEDIATING EFFECT OF PLACE ATTACHMENT ON ATTITUDE TOWARD AGING AND PSYCHOLOGICAL WELL-BEING

**DOI:** 10.1093/geroni/igad104.1183

**Published:** 2023-12-21

## Abstract

Psychological well-being has become a social challenge for vulnerable older adults and age-friendly cities/communities has been a key aging policy focusing on environmental factors to enhance well-being in Taiwan. However, how older adults perceive their living environments and whether it is important when advocating older adults’ positive attitudes toward aging leading to psychological well-being remains unknown. One of the place attachment dimensions is psychological attachment which has attracted attention in planning for aging in place and age-friendly communities (Aliakbarzadeh et al., 2021; 2022). This study aims to investigate whether psychological attachment mediated the relationship between attitudes toward aging and psychological well-being among community-dwelling older adults in Taiwan. A faced-to-faced interview of 306 individuals aged 50 and over living in the community were interviewed. The Cronbach’s alpha of all scales was over 0.70 which showed good reliability. The associations and mediation effects were tested using structural equation modeling (SEM) and the model fitted the data adequately. After controlling for sex and age, the SEM findings demonstrated that the lower level of negative attitudes toward aging were significantly and positively associated with well-being (direct effect = 0.16, p <0.05). Furthermore, the lower level of negative attitudes toward aging was positively related with place attachment and consequently predicted better psychological well-being (indirect effect = 0.048, p = 0.034; total effect = 0.209, p= 0.003). We suggest that place attachment should be monitored in the policy of age-friendly communities while improving older adults’ positive attitudes toward aging leading to a better sense of well-being.

